# Expression of transforming growth factor alpha, epidermal growth factor receptor and epidermal growth factor in precursor lesions to gastric carcinoma.

**DOI:** 10.1038/bjc.1995.7

**Published:** 1995-01

**Authors:** M. I. Filipe, M. Osborn, J. Linehan, E. Sanidas, M. J. Brito, J. Jankowski

**Affiliations:** Department of Histopathology, UMDS Guy's Hospital, London, UK.

## Abstract

**Images:**


British Journal of Cancer (1995) 71, 30-36

© 1995 Stockton Press All rights reserved 0007-0920/95 $9.00

### Expression of transforming growth factor alpha, epidermal growth factor receptor and epidermal growth factor in precursor lesions to gastric carcinoma

MI Filipe[1], M Osborn[1], J Linehan[1], E Sanidas[3], MJ Brito[2] and J Jankowski[4]

[1]*Department of Histopathology, UMDS Guy's Hospital, London, UK;* [2]*Department of Pathology, Sta Marta Hospital, Lisbon, Portugal;* [3]*Department of Surgical Oncology, Herakleion University Hospital, Crete, Greece;* [4]*ICRF, Royal Postgraduate Medical School, Du Cane Road, London, UK.*

**Summary** Epidermal growth factor (EGF), its related peptide transforming growth factor (TGF-α) and their common receptor (EGFR) have been implicated in the control of cell proliferation and differentiation in the gastrointestinal epithelium and may play an important role in gastric carcinogenesis. We compared the immunohistochemical expression and topographic distribution of these peptides using Western blot analysis in gastric carcinoma precursor lesions and in non-cancer tissue. We observed: (i) increased and extended expression of TGF-α in normal mucosa and hyperplasia in carcinoma fields compared with non-cancer controls; (ii) increased expression of EGFR in intestinal metaplasia (IM) from carcinoma fields compared with controls; (iii) EGF expression was not detected in normal mucosa and only weakly in IM; (iv) coexpression of TGF-α EGFR and EGF EGFR was higher in intestinal metaplasia in carcinoma fields than in non-cancer controls. We conclude that altered expression of TGF-α EGFR is associated with morphological changes during gastric carcinogenesis. In this regard increased expression of TGF-α is a very early event which is subsequently followed by up-regulation of EGFR and this has important biological and clinical implications.

**Keywords:** growth factors; precancer lesions; gastric carcinoma

Gastric carcinogenesis is a stepwise process which starts with molecular changes in normal cells resulting in phenotypic adaptation. In this regard chronic gastritis is one of the earliest identifiable histological abnormalities and may lead to intestinal metaplasia (IM), dysplasia and ultimately adenocarcinoma (Correa, 1988). The underlying mechanisms that control this process are not as yet fully understood. The majority of gastric carcinomas appear to be caused by environmental factors resulting in mucosal damage and repair (Parsonnet *et al.*, 1991; UK Subgroup of the ECP-Euronut-IM Study Group, 1992; Pignatelli *et al.*, 1993). This pleomorphic response is regulated in part by inhibitory and stimulatory molecules derived from proto-oncogenes and tumour-suppressor genes (Tahara, 1993). Growth factors are important regulators of cell differentiation and proliferation and play an important role in maintaining the integrity of the epithelium. Increased expression in cells or aberrant topographical distribution of the growth-regulatory peptides epidermal growth factor (EGF) and transforming growth factor α (TGF-α) and their receptor (EGFR) has been described in the gastrointestinal epithelium associated with mitogenesis and carcinogenesis (Goodlad and Wright, 1990; Jankowski, 1992; Nasim *et al.*, 1992; Filipe and Jankowski, 1993). Human TGF-α has been mapped to chromosome 2 to the short arm region 2p11-2p13, and it is known that chromosome 2 contains other genes that are involved in growth regulation and tumorigenesis. EGFR has been found to be overexpressed at high frequency in a wide range of tumours. Increased expression of TGF-α is frequent in gastric carcinoma (60%), particularly the intestinal type of carcinoma, but EGFR overexpression was less frequent (18%) in our series (Goodlad and Wright, 1990; Jain *et al.*, 1991; Lemoine *et al.*, 1991). Coexpression of EGF and its receptor (EGFR) is correlated with progression of these tumours and poor prognosis (Yonemura *et al.*, 1991, 1992).

The expression of growth factors is deregulated in invasive neoplasms but few data are available with regard to their expression in the early stages of tumorigenesis.

Correspondence: MI Filipe
Received 6 April 1994; revised 9 August 1994; accepted 17 August 1994

The purpose of this study was to assess the altered expression of growth factors EGF and TGF-α and their common receptor EGFR in the precursor lesions to gastric carcinoma and the possible role of such altered expression in the multistep process of malignancy. In addition, we were interested in assessing whether immunoreactivity to growth factors and their receptors corresponded with normal- or aberrant-sized protein products by Western blot analysis.

### Materials and methods

Formalin-fixed paraffin-embedded samples from 16 stomachs resected for early stage T1 gastric carcinoma and nine from advanced gastric carcinomas were available: 19 intestinal, 4 diffuse and two mixed (Lauren, 1965). Non cancer control biopsies were obtained from gastric ulcer or chronic gastritis patients ($n = 56$). The non-carcinoma control samples included specimens of normal histology ($n = 41$) and intestinal metaplasia ($n = 15$).

Carcinoma field changes from the 16 T1 gastric carcinomas included histologically normal mucosa ($n = 16$), hyperplasia ($n = 14$), intestinal metaplasia ($n = 17$) and dysplasia ($n = 10$, of which seven cases were high grade) (Riddel *et al.*, 1983).

Carcinoma field changes from the nine advanced gastric carcinomas included histologically normal mucosa ($n = 7$), hyperplasia ($n = 3$), intestinal metaplasia ($n = 5$) and dysplasia ($n = 4$, of which two cases were high grade).

The total numbers of carcinoma field lesions were as follows: normal, 23; intestinal metaplasia, 22; hyperplasia, 17; dysplasia, 14, of which nine lesions were high grade.

### *Immunostaining*

All material retrieved was formalin fixed and paraffin embedded from Guy's Hospital archives.

The primary antibodies used were: mouse anti-TGF-α, Ab-2, clone 213-94 (Oncogene Science), used on sections at a concentration of 0.33 $\mu g\ ml^{-1}$, overnight at 4°C; rabbit anti-EGFR, 12E (a gift from W Gullick), used on sections at a concentration of 8 $\mu g\ ml^{-1}$, for 1 h at room temperature; and rabbit anti-EGF. Ab-3 (Oncogene Science). used on sections at a concentration of $5\,\mu g\,ml^{-1}$ at room temperature for 1 h.

Three micron paraffin sections were attached to slides using poly-L-lysine (Sigma). TGF-α sections were oven dried (30 min. 60°C); EGFR and EGF were air dried. Following rehydration in phosphate-buffered saline (PBS). endogenous peroxidase activity was blocked with 0.3% hydrogen peroxide (in five parts methanol for EGFR and EGF sections). Sections were then incubated in normal goat (TGF-α), calf (EGFR) or swine (EGF) serum before incubation in the primary antibody. Subsequent incubation in biotinylated goat anti-mouse (TGF-α) or swine anti-rabbit (EGFR, EGF) serum preceded incubation with the Dako ABC-Complex kit (TGF-α). Dako streptavidin ABC kit (EGFR) or streptavidin horseradish peroxidase (EGF) (Oncogene Science). Peroxidase activity was developed with diaminobenzidine (Sigma). Slides were lightly counterstained with Carrazzi haematoxylin. For negative controls primary antibody was replaced with PBS. Positive controls were also run.

### *Interpretation of immunostaining*

This was carried out by two independent observers blind to the clinical and histological data (MIF and MO). Positive immunoreactivity for TGF-α. EGFR and EGF was found in the cell membranes and cytoplasm. Sections were only considered negative if no staining was seen in these cellular locations. For each lesion. expression of each growth factor/receptor was carried out using a semiquantitative scoring method for the following parameters: (i) intensity of staining. 0–3 for negative. weak. moderate and strong; (ii) extent of expression. 0–3 for negative. focal (up to 30% cells positive). patchy (30–50% of cells positive) or extensive (>50% cells positive); (iii) location of TGF-α. EGFR and EGF immunoreactivity within gastric glands in each lesion was assessed by dividing each gland into compartments. upper third. middle and lower third. or all three areas. upper. middle and lower. There was strong agreement in the interpretation of these parameters between the two observers (MIF and MO). and in a few cases where disagreement occurred an agreed score was achieved.

### *Statistical analysis*

The Kruskal–Wallis test was conducted to determine the different expression patterns of lesions in terms of intensity and extent of expression. This statistical test was chosen as the data were not parametric. The $Z$-values obtained are a better indicator of how the expression varied between lesions than does a mere comparison of the median expression of score values. as they take into account the range of expression between lesions of the same type. A positive score indicates that the median and range of that particular group of lesions is different when compared with the inter-group variation. The chi-squared test was also carried out to assess quantitative differences in the proportion of lesions expressing specific growth factors.

### *Western blot analysis*

Proteins were extracted from fresh-frozen specimens. including normal mucosa ($n = 3$). intestinal metaplasia ($n = 3$) and well or moderately/poorly differentiated gastric carcinomas ($n = 4$). Samples were homogenised in sodium dodecyl sulphate/glycine/Tris gel loading buffer in the presence of a protease inhibitor for 2 min. After centrifugation at 30 000 $g$ for 20 min. the resultant supernatant was frozen. The protein concentration was estimated by light spectrophotometry at 595 nm according to the Bradford method (Jankowski. 1994*a*).

Prior to running the proteins in a 15% acrylamide gel (7.5% gel was used for EGFR) at low voltage ($8\,V\,cm^{-1}$). β-mercaptoethanol was added to the samples and these were heated at 100°C for 3 min. Thirty micrograms of total protein from each sample was loaded in each gel lane.

Using a Bio-Rad trans-blot apparatus (Protean II. Bio-Rad. London. UK) the proteins were transferred from the gel to a compound nylon–nitrocellulose membrane (Satorios. London. UK). Once the transfer was completed the membrane and gel were removed and the gel was placed in Coomassie brilliant blue to assess the efficiency of the protein transfer.

The membrane was stained with Ponceau S in order to mark the molecular weight standard with a waterproof pencil. Subsequently. the membrane was fixed in 0.2% glutaraldehyde–PBS for 45 min with gentle agitation.

The membrane was then washed in PBS + 0.1% Tween. The membrane was then blocked in 3% bovine serum albumin (BSA) in PBS for 1 h. After washing. the membrane was incubated in 1:750 diluted primary antibody (Ab-2) for 2 h followed by incubations with biotinylated rabbit and anti-mouse antibody (Dako. London. UK) for 1 h and ABC peroxidase complex (Dako) ($5\,mg\,ml^{-1}$) for 30 min and DAB ($0.3\,mg\,ml^{-1}$) for 3 min. Negative control experiments included blots incubated without either the primary or the secondary antibody. and positive controls included primary breast and colonic adenocarcinomas. In addition. three paired samples of gastric tissue were incubated in plastic bags of 5 ml volume with TGF-α antibody at 1:750 dilution with or without TGF-α 50 amino acid peptide (Sigma UK) at $50\,\mu g\,ml^{-1}$.

### Results

### *Immunohistochemistry*

*Intensity of immunoreactivity* TGF-α expression was significantly greater in histologically normal mucosa from carcinoma fields than in normal control mucosa from biopsies (chi-squared test. $P<0.01$). In contrast. levels of TGF-α

**Table I** Intensity of TGF-α expression

| Intensity of staining | *Normal mucosa* Control (n = 39) | *Normal mucosa* Ca field (n = 23) | *Intestinal metaplasia* Control (n = 12) | *Intestinal metaplasia* Ca field (n = 19) | *Hyperplasia* Ca field (n = 11) | *Dysplasia* Ca field (n = 14) |
|---|---|---|---|---|---|---|
| III | ●●●● | ●●●●●<br>● | ●●● | ●● | ●●●● | ● |
| II | ●●●●●<br>●●●● | ●●●●●<br>●●●●●<br>●●● | ●●●●● | ●●●●●<br>●● | ●●● | ●●● |
| I | ●●●●●<br>●●●●●<br>●●●●●<br>●●●●●<br>●●●●● | ●●●● | ●●●● | ●●●●●<br>●●● | ●●●● | ●●●●●<br>●●● |
| 0 | ● | | | ●● | | ●● |

expression in intestinal metaplasia from carcinoma fields were similar to those seen in intestinal metaplasia from non-cancer patients (control biopsies). Dysplasia from carcinoma fields showed lower levels of TGF-α expression in both high-grade and low-grade dysplasia than in all other precursor lesions (but the numbers were too few to allow formal statistical analysis). Based on Kruskall–Wallis analysis, TGF-α expression was greatest in histological epithelium from carcinoma fields ($Z = 2.84$) and lowest in normal control specimens ($Z = -2.16$) (Table I).

EGFR expression was moderate to weak in most types of lesion, with no difference between histologically normal mucosa from carcinoma fields and non-cancer controls. However, intestinal metaplasia from carcinoma fields showed significantly higher expression of EGFR than its control ($P<0.1$; $Z = 3.8$ *vs* $Z = 1.07$) (Table II).

Most lesion types failed to show EGF expression, with the exception of intestinal metaplasia, which presented slightly higher $z$-values than all other lesions.

*Extent of immunoreactivity* TGF-α expression was extensive in intestinal metaplasia in both carcinoma fields and non-cancer controls. Extensive immunoreactivity was also seen in hyperplasia and normal mucosa in carcinoma fields but was patchy compared with the corresponding controls ($P<0.1$). A similar trend was seen with EGFR expression. In dysplastic mucosa both TGF-α and EGFR staining was predominantly patchy/focal. The pattern of EGF expression was dwarfed by the high rate of negative cases, but when detected it tended to be patchy in all lesion types.

*Positional TGF-α and EGFR immunoreactivity within gland compartments* The positional expression patterns of precursor lesions and controls were very clear for TGF-α and EGFR (Tables III and IV). In the majority of normal mucosas from non-cancer control biopsies, TGF-α immunoreactivity was confined to the upper gland compartment (95%) compared with only 22% of normal mucosas in carcinoma fields (Figures 1 and 2). None of the normal control biopsies expressed TGF-α throughout the whole glandular epithelium compared with 77% of 'normal' mucosa from carcinoma fields. These differences between distribution of TGF-α in the upper gland compartment are less marked for intestinal metaplasia, being 64% and 77% in IM in carcinoma field lesions and IM non-cancer controls respectively. Most dysplastic lesions had further reductions in TGF-α in the upper gland compartment (58%).

The positional patterns of EGFR expression are similar to but more marked than those revealed with TGF-α. In particular, an extensive EGFR expression throughout the whole glandular epithelium is seen in intestinal metaplasia in carcinoma fields (80%) compared with controls (50%) (Figures 3 and 4). In addition, the intensity of EGFR expression increases serially in normal gastric mucosa from controls to normal in gastric cancer fields, intestinal metaplasia and intestinal metaplasia from carcinoma stomachs and to dysplasia. In this regard a specimen of dysplastic mucosa with IM showed markedly increased EGFR expression (Figure 5).

EGF was not immunohistochemically detectable in normal mucosa but was weakly detectable in intestinal metaplasia.

*Coexpression* High levels of TGF-α/EGFR coexpression were observed in the precursor lesions with the exception of hyperplasia. In particular, the median intensity of immunoreactivity was twice as high in normal mucosa from carcinoma fields as in non-cancer controls, but no difference was detected in intestinal metaplasia. Conversely, in the few speci-

**Table II** Intensity of EGFR expression

| *Intensity of staining* | *Normal mucosa* Control (n = 41) | *Normal mucosa* Ca field (n = 20) | *Intestinal metaplasia* Control (n = 15) | *Intestinal metaplasia* Ca field (n = 22) | *Hyperplasia* Ca field (n = 17) | *Dysplasia* Ca field (n = 13) |
|---|---|---|---|---|---|---|
| III | ● | | | ●●●●● | ● | ● |
| II | ●●●●●<br>●●● | ●●●●● | ●●●●●<br>●●● | ●●●●●<br>●●●●●<br>●●●● | ● | ●●● |
| I | ●●●●●<br>●●●●●<br>●●●●●<br>●●●●●<br>●●●●● | ●●●●●<br>●●●● | ●●●●●<br>●● | ●● | ●●●●● | ●●●●●<br>● |
| 0 | ●●●●●<br>●● | ●●●●●<br>● | | ● | ●●●●●<br>●●●●● | ●●● |

**Table III** Positional expression of TGF-α

| *Zone showing expression* | *Normal mucosa* Control (n = 39) | *Normal mucosa* Ca field (n = 22) | *Intestinal metaplasia* Control (n = 13) | *Intestinal metaplasia* Ca field (n = 14) | *Hyperplasia* Ca field (n = 12) | *Dysplasia* Ca field (n = 12) |
|---|---|---|---|---|---|---|
| Top + middle + lower | | ●●●●●<br>●●●●●<br>●●●●●<br>●●<br>(77%) | ●●●<br>(23%) | ●●●●●<br>(36%) | ●●●●●<br>●<br>(50%) | ●●●●<br>(33%) |
| Middle + lower | ●●<br>(5%) | | | | ●●●<br>(25%) | ● |
| Upper gland<br>Surface + foveolar epithelium | ●●●●●<br>●●●●●<br>●●●●●<br>●●●●●<br>●●●●●<br>●●●●●<br>●●●●●<br>●●<br>(95%) | ●●●●●<br>(22%) | ●●●●●<br>●●●●●<br>(77%) | ●●●●●<br>●●●●<br>(64%) | ●●●<br>(25%) | ●●●●●<br>●●<br>(58%) |

**Table IV** Positional expression of EGFR

| Zone showing expression | Normal mucosa Control (n = 34) | Normal mucosa Ca field (n = 15) | Intestinal metaplasia Control (n = 12) | Intestinal metaplasia Ca field (n = 20) | Hyperplasia Ca field (n = 8) | Dysplasia Ca field (n = 8) |
|---|---|---|---|---|---|---|
| Top + middle + lower | ●●● (9%) | ●●●●● (33%) | ●●●●● ● (50%) | ●●●●● ●●●●● ●●●●● ● (80%) | ●●●●● (63%) | ●●●●● ● (75%) |
| Middle + lower | | ● (7%) | | ● (5%) | ● (12%) | |
| Upper gland Surface + foveolar epithelium | ●●●●● ●●●●● ●●●●● ●●●●● ●●●●● ●●●●● ● (91%) | ●●●●● ●●●● (60%) | ●●●●● ● (50%) | ●●● (15%) | ●● (25%) | ●● (25%) |

**Figure 1** TGF-α expression in normal gastric epithelium. Bar = 50 μm.

**Figure 2** TGF-α extended expression in 'normal' gastric epithelium in carcinoma fields. Bar = 100 μm.

**Figure 3** EGFR expression in intestinal metaplasia in non-cancer field. Bar = 50 μm.

mens which had EGF/EGFR coexpression, this was similar in normal mucosa from carcinoma fields and non-cancer controls but was higher in intestinal metaplasia from carcinoma fields than in their counterparts in non-cancer controls.

*Western blot analysis* EGF immunoreactivity corresponded with molecular weight species of 6 kDa, however larger molecular weight bands were also present at 9, 14 and 18 kDa. This may indicate a common epitope of the EGF antibody with EGF and its precursors or other related members of the EGF family (Figure 6).

TGF-α immunoreactivity was strongest at 6 kDa (Figure 7). It is also evident that the signet ring carcinoma has reduced immunoreactivity compared with the well-differentiated carcinomas and the intestinal metaplasia. In addition, a competition reaction was performed in which prior incubation of TGF-α (TGF-α 50 amino acid peptide, Sigma, UK) markedly reduced the subsequent immunoreactivity in the Western blot compared with unblocked paired specimens (Figure 8).

EGFR immunoreactivity was maximal at 170 kDa, indicating specificity for the mature receptor (Figure 9).

### Discussion

### *Expression of EGF, TGF-α and EGFR*

Our data support the hypothesis that increased EGFR and TGF-α expression is associated with early events in gastric

**Figure 4** EGFR extended expression in intestinal metaplasia in carcinoma field. Bar = 50 μm.

**Figure 5** EGFR extended expression in dysplastic epithelium. Bar = 50 μm.

**Figure 6** EGF has multiple immunoreactive bands between 6 and 14 kDa suggesting cross-reaction with EGF and its precursors. A, Carcinoma; B, Intestinal metaplasia; C, NAD.

**Figure 7** TGF-α corresponds to 6 kDa in carcinoma and intestinal metaplasia (IM). All specimens have similar immunoreactivity except the poorly differentiated (signet ring) carcinoma, which shows markedly reduced staining. a and b, carcinoma; c and d, intestinal metaplasia; e, poorly differentiated carcinoma.

tumorigenesis. Altered expression of TGF-α and EGFR in terms of greater intensity of immunostaining and extended involvement of the glandular epithelium occurred at an earlier stage in histologically normal mucosa and glandular hyperplasia in carcinoma fields than in non-cancer controls.

Up-regulation of TGF-α appears to be one of the earliest events during gastric tumorigenesis as it is induced in normal mucosa adjacent to cancer. Subsequently, EGFR expression is up-regulated during the generation of metaplastic epithelium.

*Mechanisms of growth factor-enhanced tumorigenesis*

Growth factors are components of signal transduction pathways which have a considerable spectrum of biological

**Figure 8** TGF-α immunoreactivity in three paired specimens of gastric tissue intestinal metaplasia and gastric carcinoma with (**a**) and without (**b**) incubation with 50 μg of TGF-α peptide during immunostaining. It is clear that the TGF-α peptide markedly reduces the immunoreactivity.

**Figure 9** EGFR shows strong immunoreactivity bands at 160–170 kDa for well-differentiated adenocarcinoma and weaker bands for intestinal metaplasia. A and B, carcinoma; C and D, intestinal metaplasia.

activity, such as control of cell proliferation, differentiation, apoptosis, transformation and neovascularisation. In addition, EGF and TGF-α may regulate the transition rate between $G_2$-phase and mitosis of the cell cycle. TGF-α has a greater mitogenic effect than EGF and a longer duration of action (Di Marco *et al.*, 1990; Jankowski, 1994*b*).

In this study the increased expression of TGF-α and EGFR in precancerous lesions correlates with observed higher indices of proliferation in these lesions. In particular, a shift in the compartmental position of the proliferating cells to the upper compartment or throughout the gland was noted in areas of dysplasia and intestinal metaplasia of the incomplete type 3, which is associated with a higher increased risk of developing carcinoma than the complete type IM (Rokkas *et al.*, 1991; Filipe *et al.*, 1993, 1994).

In addition, the increased coexpression of EGFR/TGF-α in precancer stages suggest an aberrant autocrine loop which may play a role in malignant transformation. Our data and those of others indicate that the events leading to malignancy follow a pathway initiated by microenvironmental damage, leading to gastritis and a resistant metaplastic epithelium that expresses TGF-α strongly. A combination of ligand and receptor, such as TGF-α and EGFR, may subsequently lead the cells to enter a final common pathway of oncogene expression that results in breakdown of the normal feedback control, leading ultimately to neoplasia and particularly the intestinal type gastric carcinoma.

It is possible that the various stages of precancer are under the influence of different mechanisms of growth regulation:

(i) An inappropriate increase in functional molecules such as EGFR at an early stage may lead to an enhanced production of EGFR protein without negative feedback inhibition.
(ii) A change in autocrine or paracrine growth regulation at an early/intermediate stage, may occur allowing TGF-α from one cell to bind to EGFR on nearby cells; however, the expression of TGF-α and EGFR would have to be very high to counteract the effect of dilution of the ligand in the extracellular space.
(iii) At a late stage synergism between growth-regulatory molecules may stimulate cell proliferation and other oncogenes such as c-*fos* and c-*myc*, as described in other gastrointestinal tumours (Di Marco *et al.*, 1990; Tahara, 1990; Jankowski, 1994*b*).

*Expression of additional growth factors*

Other factors may also be involved in the process of gastric carcinogenesis, and some may act at an early stage. (Tahara,

1990, 1993; Martin *et al.*, 1992; Tohdo *et al.*, 1993; Brito *et al.*, 1994; Jankowski, 1994*b*). Overexpression of Tpr-met RNA has been detected in the earliest stage of superficial gastritis with hyperplasia and is also present with variable intensity in the various stages of progression to carcinoma, suggesting the possible involvement of this oncogene in gastric tumorigenesis (Soman *et al.*, 1991).

Moreover, *crypto*, a gene of the EGF family, is overexpressed in intestinal metaplasia as well as in well-differentiated adenocarcinoma. Also of interest is that the 2.2 kb mRNA is detected in almost all intestinal metaplasias and well-differentiated adenocarcinomas (Tahara, 1993). These findings indicate that different genetic pathways of stomach carcinogenesis may exist for well-differentiated and poorly differentiated carcinomas. Some of the intestinal type carcinomas may develop by a cumulative series of genetic alterations similar to those that occur in colorectal cancer. The exciting message is that increased knowledge of the molecular events occurring in the precancer stage may have important implications for the early detection of cancer risk and for its prevention.

*Conclusions and serial expression of growth factors during tumorigenesis*

Growth factors play an important role in the early stages and progression of gastric carcinogenesis. Our data suggest a potential role for quantitative/qualitative changes in growth factors in gastric carcinogenesis. A possible hypothesis of chronology of events could be as follows:

(i) $\uparrow$ TGF-α and Tpr-met during field defect.
(ii) $\uparrow$ EGFR associated with the metaplastic phenotype.
(iii) $\uparrow$ TGF-α and EGFR with dysplasia.
(iv) $\uparrow$ c-*erb*B2 with generation of invasive neoplasia.
(v) $\uparrow$ EGF with advanced neoplasia and metastases.

**References**

BRITO MJ, WILLIAMS GT, THOMPSON H AND FILIPE MI. (1994). Expression in p53 early (T1) gastric carcinoma and precancerous adjacent mucosa. *Gut*, (in press).

CORREA P. (1988). A human model of gastric carcinogenesis. *Cancer Res.*, **48**, 3554-3560.

DI MARCO E, PIERCE JH, AARONSON SA AND DIFIORE PP. (1990). Mechanisms by which EGF receptor and the TGFalpha contribute to malignant transformation. *Nat. Immun. Cell Growth Regul.*, **9**, 209-221.

FILIPE MI AND JANKOWSKI J. (1993). Growth factors and oncogenes in Barrett's oesophagus and gastric metaplasia. *Endoscopy*, **25** (Suppl.), 637-641.

FILIPE MI, MENDES R, LANE DP AND MORRIS RW. (1993). Assessment of cell proliferation in precursor stages of gastric carcinoma using the PC10 antibody to PCNA. *Histopathology*, **22**, 349-354.

FILIPE MI, MUNOZ N, KATO I, POMPE-KIRN V, JUTERSEK A, TEUCHMANN S, BENZ M, PRISON T AND MATKO I. (1994). Intestinal metaplasia types and the risk of gastric cancer: a cohort study in Slovenia. *Int. J. Cancer*, **57**, 324-329.

GOODLAD RA AND WRIGHT NA. (1990). Growth control factors in the gastrointestinal tract. In: *Ballieres Clinical Gastroenterology*, Ciclitira PJ (ed.) pp. 143-158. Balliére Tindall: London.

JAIN S, FILIPE MI, GULLICK WJ, LINEHAN J AND MORRIS RW. (1991). C-erbB-2 proto-oncogene expression and its relationship to survival in gastric carcinoma: an immunohistochemical study on archival material. *Int. J. Cancer*, **48**, 668-671.

JANKOWSKI J. (1994*a*). *Gene Analysis and Manipulation*, Jankowski J and Polak J (eds). University Press: London.

JANKOWSKI J. (1994*b*). Altered gene expression of growth factors and their receptors during oesophageal tumorigenesis. *Gastroenterol. Clin. Biol.*, **18**, D40-D45.

JANKOWSKI J, AL-RAWI HJ, JOHNSTON DA, HOPWOOD D, FILIPE MI, COGHILL G AND WORMSLEY KG. (1992). Growth regulatory peptides in gastric mucosa. *Clin. Sci.*, **82**, 581-587.

LAUREN P. (1965). The two histological main types of gastric carcinoma. Diffuse and so-called intestinal type carcinoma. An attempt at histoclinical classification. *Acta Pathol. Microbiol. Scand.*, **64**, 31-49.

LEMOINE NR, JAIN S, SILVESTRE F, LOPES C, HUGHES CM, McLELLAND E, GULLICK WJ AND FILIPE MI. (1991). Amplfication and overexpression of the EGF receptor and c-erbB-2 proto-oncogenes in human stomach cancer. *Br. J. Cancer*, **64**, 79-83.

MARTIN HM, FILIPE MI, MORRIS RW, LANE DP AND SILVESTRE F. (1992). p53 expression and prognosis in gastric carcinoma. *Int. J. Cancer*, **50**, 859-862.

NASIM MM, THOMAS DM, ALISON MR AND FILIPE MI. (1992). Transforming growth factor alpha expression in normal mucosa, intestinal metaplasia, dysplasia and gastric carcinoma - an immunohistochemical study. *Histopathology*, **20**, 339-343.

PARSONNET J, FRIEDMAN GD, VANDERSTEEN DP, CHANG Y, VOGELMAN JH, ORENTREICH N AND SIBLEY RK. (1991). Helicobacter pylori infection and the risk of gastric cancer. *N. Engl. J. Med.*, **325**, 1127-1131.

PIGNATELLI B, MALAVEILLE C AND BARTOCH H. (1993). Intragastric mutagens and lowered anti-oxidant defence as risk factors for gastric cancer. *Eur. J. Cancer Prev.*, **2** (Suppl. 2), 9-11.

RIDDEL RN, GOLDMAN H, RANSOHOFF DF, HAMILTON SR, MORSON BC, SOMMERS SC AND YARDLEY JH. (1983). Dysplasia in inflammatory bowel disease: standardized classification with provisional clinical implication. *Hum. Pathol.*, **14**, 931-969.

ROKKAS T, FILIPE MI AND SLADEN GE. (1991). Detection of an increased incidence of early gastric cancer in patients with intestinal metaplasia type III who are closely followed up. *Gut*, **32**, 1110-1113.

SOMAN NR, CORREA P, RUIZ BA AND WOGAN GN. (1991). The TPR-MET oncogenic rearrangement is present and expressed in human gastric carcinoma and precursor lesions. *Proc. Natl Acad. Sci. USA*, **88**, 4892-4896.

TAHARA E. (1990). Growth factors and oncogenes in human gastrointestinal cancer. *J. Cancer Res. Clin. Oncol.*, **45**, 121-131.

TAHARA E. (1993). Molecular mechanisms of stomach carcinogenesis. *J. Cancer Res. Clin. Oncol.*, **119**, 265-272.

TOHDO H, YOKOZAKI H, HARUMA K, KAJIYAMA G AND TAHARA E. (1993). p53 gene mutations in gastric adenomas. *Virchows Archiv. B. Cell. Pathol.*, **63**, 191-195.

UK SUBGROUP OF THE ECP-EURONUT-IM STUDY GROUP (1992). Plasma vitamin concentration in patients with intestinal metaplasia and in controls *Eur. J. Cancer Prev.*, **1**, 177-186.

YONEMURA Y, SUGIYAMA K, FUSHIDA S, KAMATA T, OHUYAMA S, KIMURA H, YAMAGUCHI A AND MIYAZAKI I. (1991). Tissue status of epidermal growth factor and its receptor as an indicator of poor prognosis in patients with gastric cancer. *Anal. Cell. Pathol.*, **3**, 343-350.

YONEMURA Y, TAKAMURA H, NINOMIYA I, FUSHIDA S, TSUGAWA K, KAJI M, NAKAI Y, OHOYAMA S, YAMAGUCHI A AND MIYAZAKI I. (1992). Interrelationship between transforming growth factor - alpha and epidermal growth factor receptor in advanced gastric cancer. *Oncol.*, **49**, 157-161.


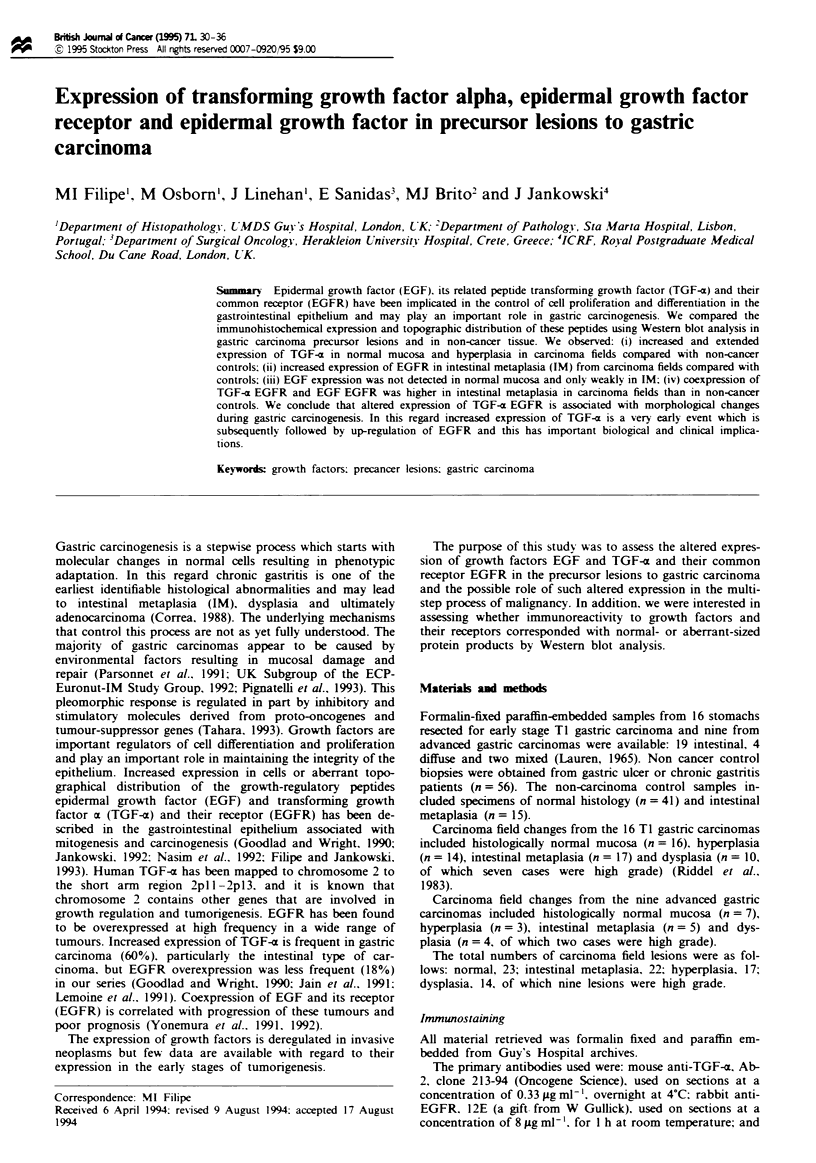

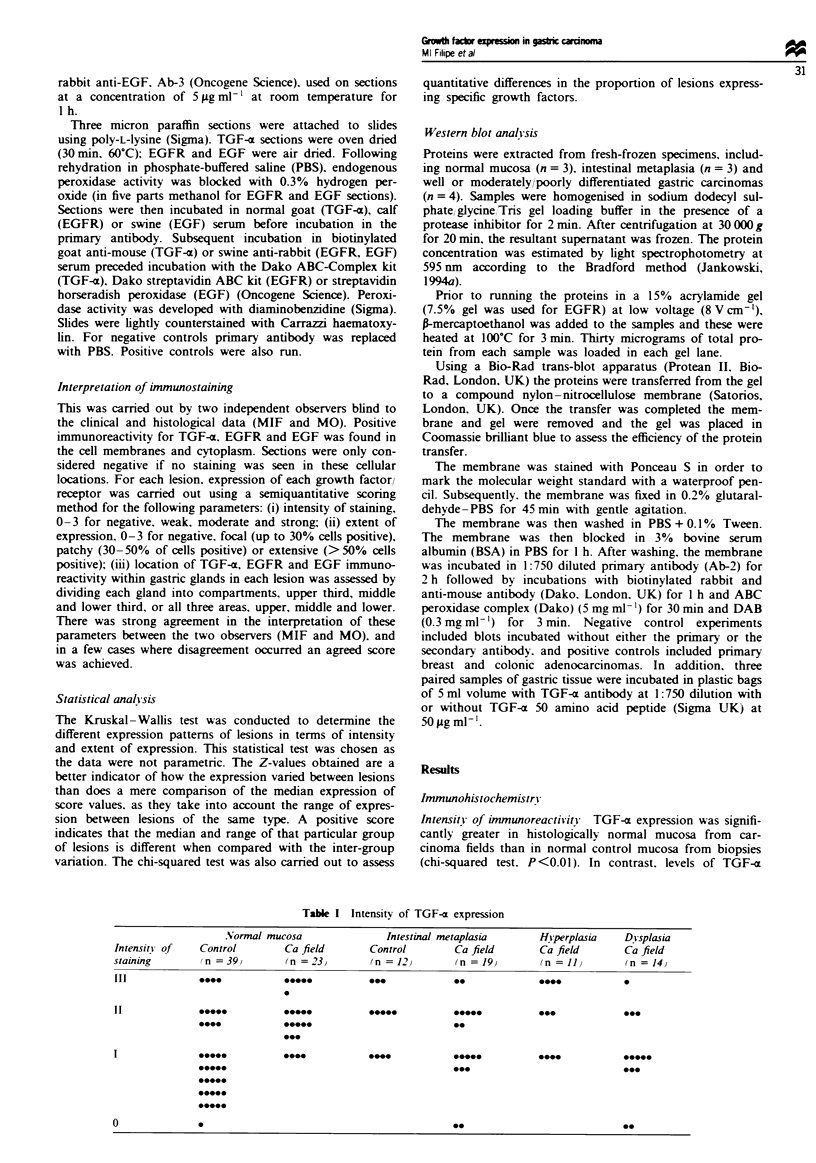

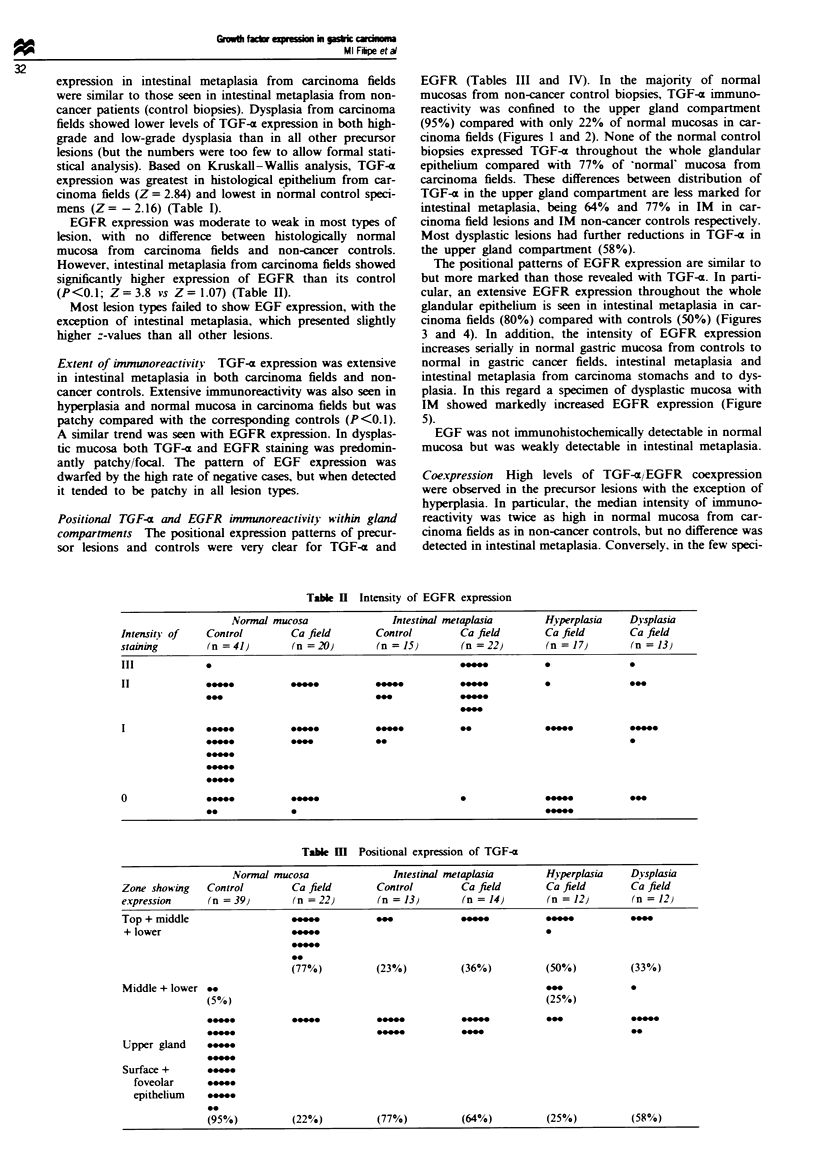

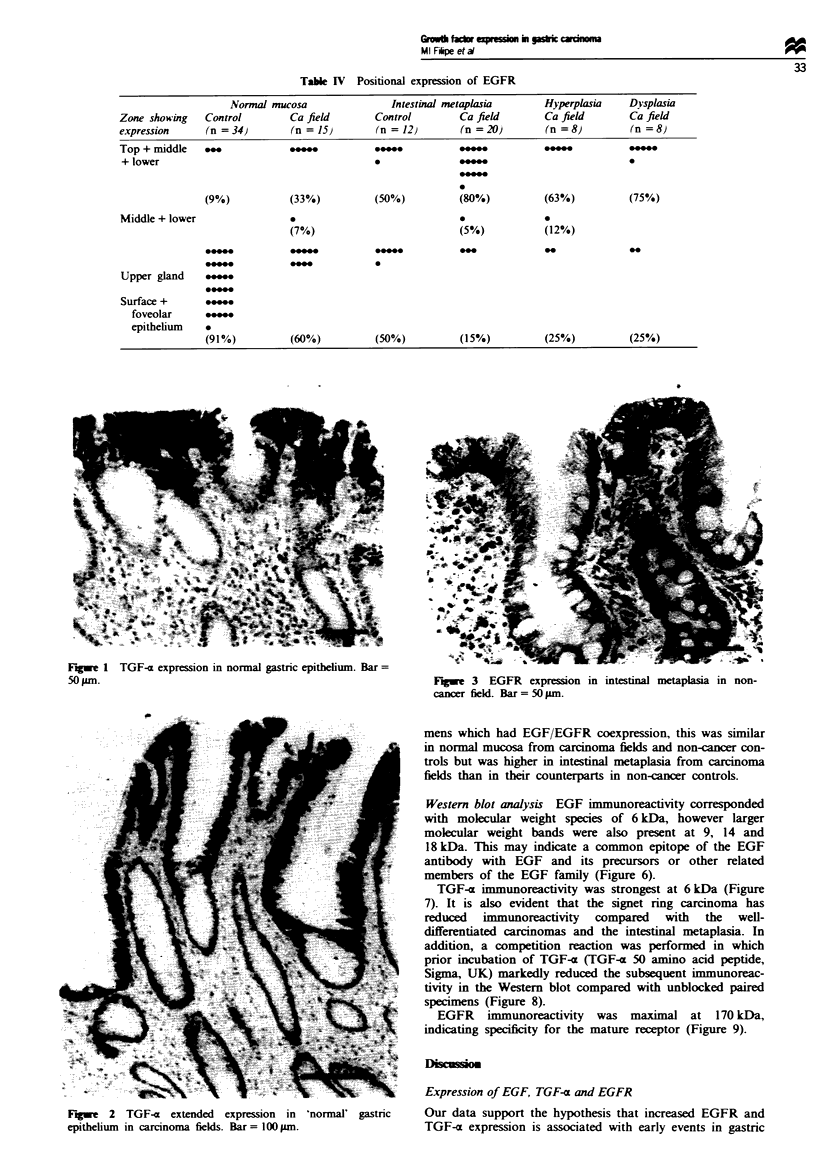

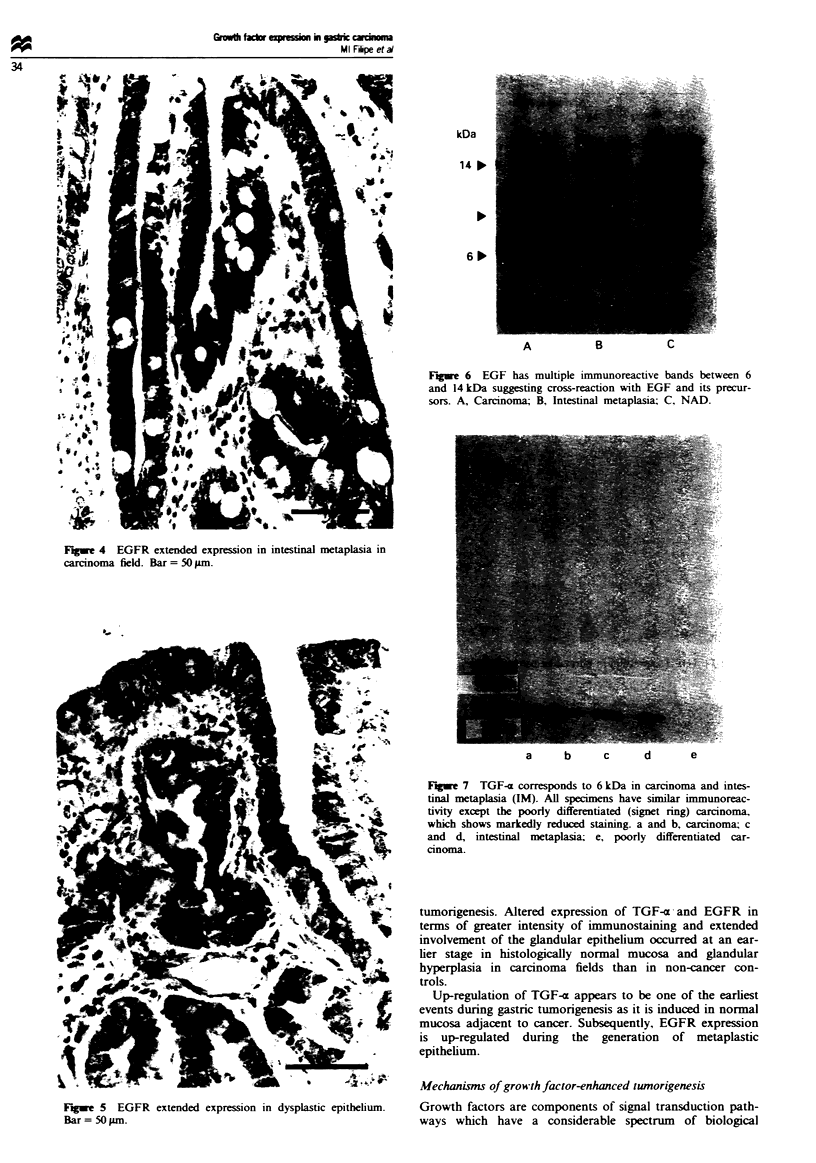

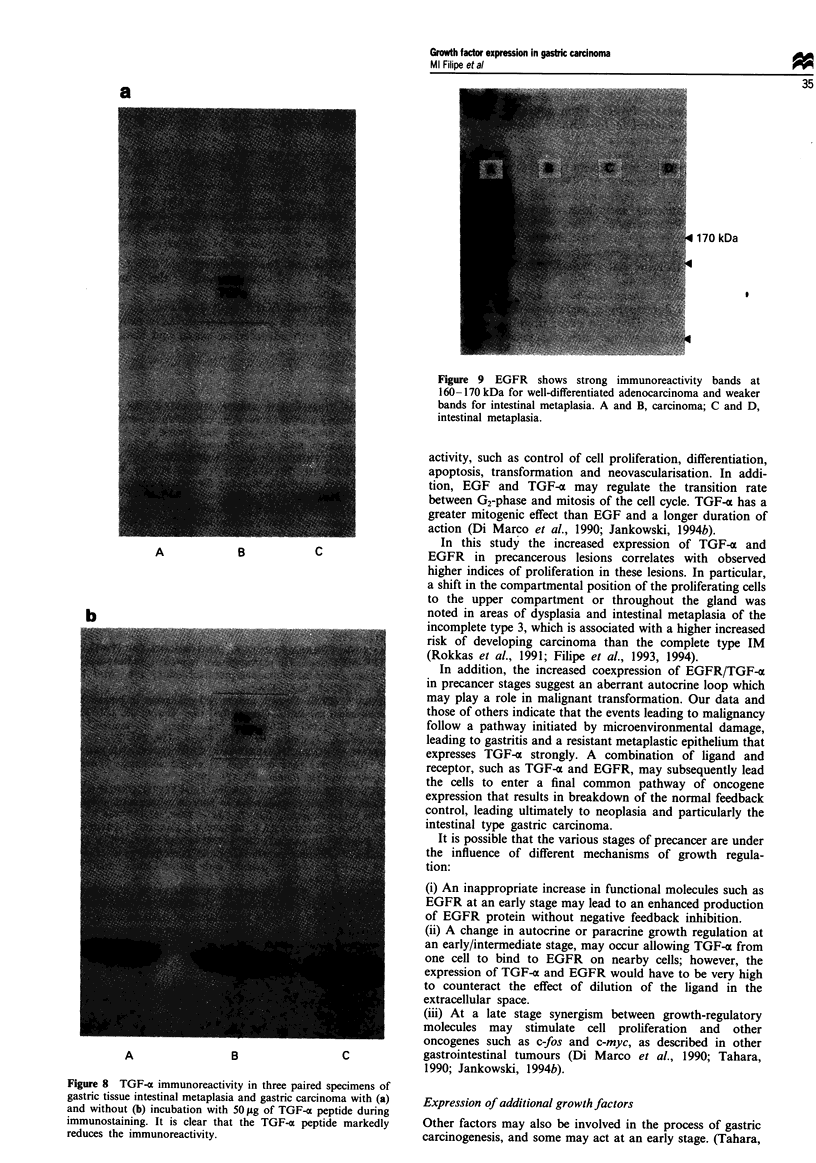

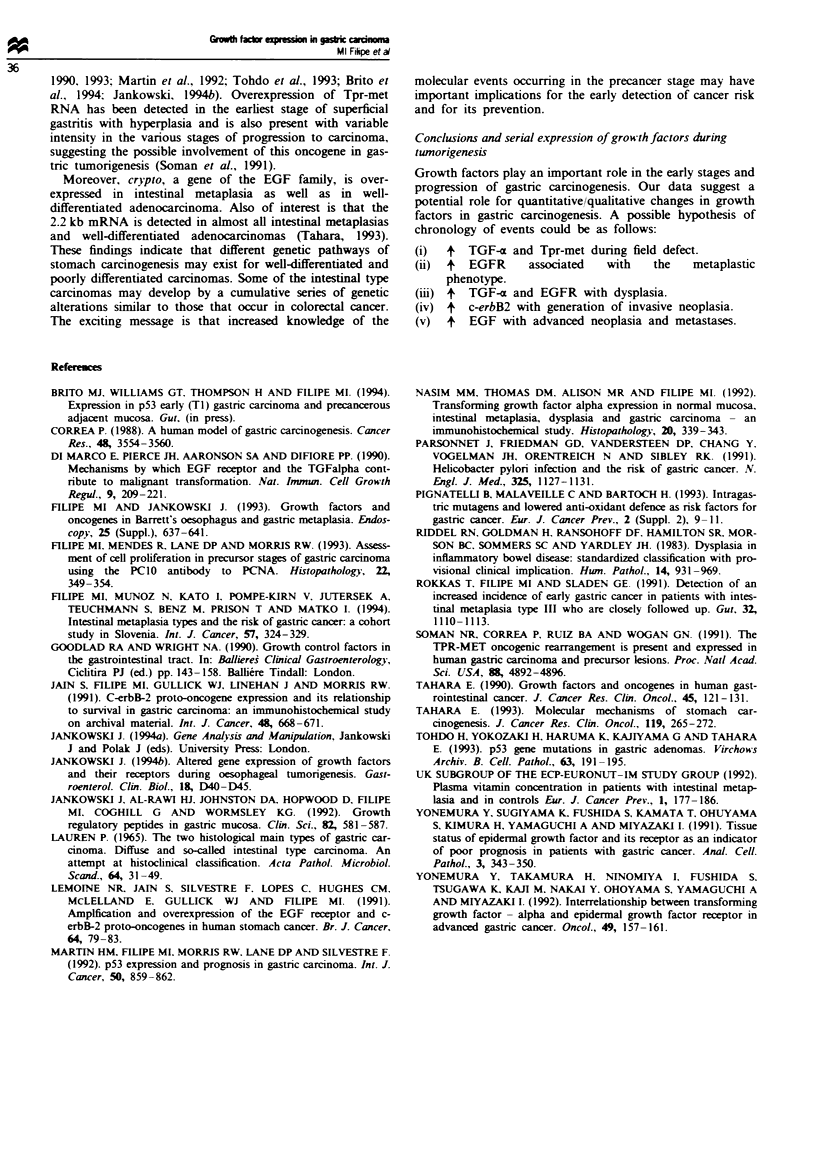


## References

[OCR_00717] Correa P. (1988). A human model of gastric carcinogenesis.. Cancer Res.

[OCR_00721] Di Marco E., Pierce J. H., Aaronson S. A., Di Fiore P. P. (1990). Mechanisms by which EGF receptor and TGF alpha contribute to malignant transformation.. Nat Immun Cell Growth Regul.

[OCR_00727] Filipe M. I., Jankowski J. (1993). Growth factors and oncogenes in Barrett's oesophagus and gastric metaplasia.. Endoscopy.

[OCR_00732] Filipe M. I., Mendes R., Lane D. P., Morris R. W. (1993). Assessment of proliferating cell nuclear antigen expression in precursor stages of gastric carcinoma using the PC10 antibody to PCNA.. Histopathology.

[OCR_00739] Filipe M. I., Muñoz N., Matko I., Kato I., Pompe-Kirn V., Jutersek A., Teuchmann S., Benz M., Prijon T. (1994). Intestinal metaplasia types and the risk of gastric cancer: a cohort study in Slovenia.. Int J Cancer.

[OCR_00749] Jain S., Filipe M. I., Gullick W. J., Linehan J., Morris R. W. (1991). c-erbB-2 proto-oncogene expression and its relationship to survival in gastric carcinoma: an immunohistochemical study on archival material.. Int J Cancer.

[OCR_00757] Jankowski J. (1994). Altered gene expression of growth factors and their receptors during esophageal tumorigenesis.. Gastroenterol Clin Biol.

[OCR_00764] Jankowski J., al-Rawi H. J., Johnston D. A., Hopwood D., Filipe M. I., Coghill G., Wormsley K. G. (1992). Growth regulatory peptides in gastric mucosa.. Clin Sci (Lond).

[OCR_00769] LAUREN P. (1965). THE TWO HISTOLOGICAL MAIN TYPES OF GASTRIC CARCINOMA: DIFFUSE AND SO-CALLED INTESTINAL-TYPE CARCINOMA. AN ATTEMPT AT A HISTO-CLINICAL CLASSIFICATION.. Acta Pathol Microbiol Scand.

[OCR_00775] Lemoine N. R., Jain S., Silvestre F., Lopes C., Hughes C. M., McLelland E., Gullick W. J., Filipe M. I. (1991). Amplification and overexpression of the EGF receptor and c-erbB-2 proto-oncogenes in human stomach cancer.. Br J Cancer.

[OCR_00782] Martin H. M., Filipe M. I., Morris R. W., Lane D. P., Silvestre F. (1992). p53 expression and prognosis in gastric carcinoma.. Int J Cancer.

[OCR_00787] Nasim M. M., Thomas D. M., Alison M. R., Filipe M. I. (1992). Transforming growth factor alpha expression in normal gastric mucosa, intestinal metaplasia, dysplasia and gastric carcinoma--an immunohistochemical study.. Histopathology.

[OCR_00794] Parsonnet J., Friedman G. D., Vandersteen D. P., Chang Y., Vogelman J. H., Orentreich N., Sibley R. K. (1991). Helicobacter pylori infection and the risk of gastric carcinoma.. N Engl J Med.

[OCR_00799] Pignatelli B., Malaveille C., Bartsch H. (1993). Intragastric mutagens and lowered anti-oxidant defence as risk factors for gastric cancer.. Eur J Cancer Prev.

[OCR_00804] Riddell R. H., Goldman H., Ransohoff D. F., Appelman H. D., Fenoglio C. M., Haggitt R. C., Ahren C., Correa P., Hamilton S. R., Morson B. C. (1983). Dysplasia in inflammatory bowel disease: standardized classification with provisional clinical applications.. Hum Pathol.

[OCR_00808] Rokkas T., Filipe M. I., Sladen G. E. (1991). Detection of an increased incidence of early gastric cancer in patients with intestinal metaplasia type III who are closely followed up.. Gut.

[OCR_00814] Soman N. R., Correa P., Ruiz B. A., Wogan G. N. (1991). The TPR-MET oncogenic rearrangement is present and expressed in human gastric carcinoma and precursor lesions.. Proc Natl Acad Sci U S A.

[OCR_00822] Tahara E. (1990). Growth factors and oncogenes in human gastrointestinal carcinomas.. J Cancer Res Clin Oncol.

[OCR_00824] Tahara E. (1993). Molecular mechanism of stomach carcinogenesis.. J Cancer Res Clin Oncol.

[OCR_00830] Tohdo H., Yokozaki H., Haruma K., Kajiyama G., Tahara E. (1993). p53 gene mutations in gastric adenomas.. Virchows Arch B Cell Pathol Incl Mol Pathol.

[OCR_00838] Yonemura Y., Sugiyama K., Fushida S., Kamata T., Ohoyama S., Kimura H., Yamaguchi A., Miyazaki I. (1991). Tissue status of epidermal growth factor and its receptor as an indicator of poor prognosis in patients with gastric cancer.. Anal Cell Pathol.

[OCR_00845] Yonemura Y., Takamura H., Ninomiya I., Fushida S., Tsugawa K., Kaji M., Nakai Y., Ohoyama S., Yamaguchi A., Miyazaki I. (1992). Interrelationship between transforming growth factor-alpha and epidermal growth factor receptor in advanced gastric cancer.. Oncology.

